# Molecular phylogeny of 16S rRNA sequences from Ugba (Pentaclethra macrophylla) seeds

**DOI:** 10.1099/acmi.0.001060.v3

**Published:** 2026-07-17

**Authors:** Ogueri Nwaiwu, Helen Onyeaka, Vincent Ifeanyi Ibekwe, Sylvester Ifunanya Okorondu, Justin Chikezie Nnokwe, Kelechi Chikanma Edward, Paul Chidoka Chikezie, Ihuoma Uzoamaka Offor-Emenike, Nicholas Chinedu Ewelike, Nnaemeka Julian Anyanwu, Ikenna Ndubuisi Nwachukwu, Etienne Chukwuma Chinakwe, Monica Mmachukwu Okorondu

**Affiliations:** 1 School of Chemical Engineering, University of Birmingham, Edgbaston Park, Birmingham, B15 2TT, UK; 2 Department of Microbiology, Federal University of Technology, Owerri, Nigeria; 3 Department of Biology, Federal University of Technology, Owerri, Nigeria; 4 Department of Microbiology, Michael Okpara University of Agriculture, Umudike, Nigeria; 5 Department of Biochemistry, Imo State University, Owerri, Nigeria; 6 School of Sciences, Coventry University, England, CV1 2DS, UK; 7 Department of Biology, Alvan Ikoku Federal University of Education, Owerri, Nigeria; 8 Department of Animal Science and Technology, Federal University of Technology, Owerri, Nigeria; 9 Department of Biochemistry, Federal University of Technology, Owerri, Nigeria

**Keywords:** ancestral sequences, evolution, food safety, *Ugba*, *Pentaclethra macrophylla*, phylogeny

## Abstract

The evolutionary analysis of bacterial species harbouring 16S rRNA sequences detected in the oil bean seeds of *Ugba* (*Pentaclethra macrophylla*) was carried out. The food product has a high socio-economic relevance to communities where it is consumed. Species such as *Kurthia gibsonii*, *Stenotrophomonas geniculata* and *Alcaligenes nematophilus* found in Ugba may have occurred in the environment and entered the sample in the field during or before harvest. The phylogenetic analysis of 35 sequences showed that some strains of the same species resolved into different monophyletic groups, suggesting species divergence or distinct evolutionary lineages. The species *K. gibsonii* was found to be the earliest ancestor following sequence-based ancestral analysis, suggesting that it was present in the analysed samples before other bacteria. The *Ugba* seeds appear to harbour a diverse group of bacteria and will benefit from metagenomic investigations as well as studies of the mechanism of survival and succession to reveal the true nature of the resident flora. This will help safeguard public health and highlight the organism’s relevance to food safety surveillance and microbial evolution. Increased knowledge of the resident organisms will also lead to the improvement of fermentation techniques and enhance the quality of the final product.

**Impact Statement**

This study highlights that *Ugba* may be beneficial or risky and all stakeholders should join hands to unravel when it can change from a delicacy to a death agent. This study will help galvanize more action on understanding resident flora from farm to fork and initiate the introduction of modern food safety practices in its preparation, storage and sale. It is also hoped that this study will initiate studies on the biochemical constituents at harvest and point of sale and the effect of different processing methods on pathogenic and non-pathogenic microbes and overall toxicity. In the long run, it is hoped that fatalities will no longer be reported after consumption.

## Data Summary

All data associated with this work are reported within the article, and sequences were deposited in GenBank under the accession numbers PQ865836–PQ865870.

## Introduction

The seeds of the oil bean (*Pentaclethra macrophylla*), known as *Ugba* or *Ukpaka* in South-Eastern Nigeria, are consumed by many people in the whole of that region. It can be eaten on its own or used as a food condiment and can be used to make several food delicacies [1]. The seed has a very high socio-economic and cultural importance. It is a common delicacy served at traditional ceremonies and generates income for traders. The preparation of the product has been described by other investigators [2,3]. Briefly, the seeds are boiled for hours, after which spontaneous alkaline fermentation is carried out from half a day to a few days. The well-fermented seeds are then sliced into thin or thick slices, depending on preference and then packed in dry banana leaves for sale. The taste and texture of the seeds may vary. Also, the preparation and the finished product may depend on community’s history. The variation in texture may be due to the tree from which the seeds are obtained. The genetic diversity of *Ugba* trees has been elucidated [4] using the *rbcl* gene, and the data showed that there were variations in the guanine-cytosine (GC%) content of different tree accessions.

The micro-organisms associated with the seeds of *Ugba* may provide a beneficial probiotic and medicinal effect. The probiotic potential due to the micro-organisms present in the product has been reported [5] in a review. In that report, two investigations using human subjects showed the beneficial effects of *Ugba* as a probiotic. In the first investigation [6], 20 people ate *Ugba* as the only fermented product for 3 weeks, while the second one [7] used 165 subjects who ate the product for 2 weeks. In both studies, the recovered stools from participants showed an increased count of *Lactobacillus* species associated with probiotic qualities. In the past, the focus of probiotic organisms was on dairy products. *Ugba* is a non-dairy source, and recently, it has been noted that probiotic micro-organisms from non-dairy food sources are becoming increasingly important [8]. *Ugba* is also believed to be rich in polyphenols and has medicinal qualities from folklore and trials conducted with rats. The product has been reported to have bioactive compounds [9,10], and it is believed to be active against sickle cell, high cholesterol and cancer [11]. Also, compounds present in seed pods may promote wound healing [12]. Furthermore, the seeds may have antioxidant potentials [13] and could mitigate memory loss [14].

Despite the nutritional and socioeconomic importance, a report [15] highlighted that there have been reported cases of fatalities after consumption of *Ugba*. The specific cause of death was not established in many instances since no post-mortem enquiries were carried out to establish if the fatalities were due to food-borne pathogens or chemical toxicants from the seeds. In over a century of consumption of the product, anecdotal evidence suggests that the processing steps are enough to break down toxicants and prevent the proliferation of foodborne pathogens. Hence, fatalities may be due to post-process contamination with microbes during packaging or incomplete fermentation that allowed the presence of toxicants. It is instructive to note that neither the former nor the latter has been properly investigated and reported; hence, an opportunity for definitive empirical research exists to establish any aspect of the production, storage and sales of the product that may be deleterious to human health.

In the literature, pathogens of food safety concern in *Ugba* have been reported after cultural methods were used, but the reports of molecular studies on bacteria that are associated with *Ugba* seeds are scarce. There are few whole genome reports of organisms from the product or characterization of 16S rRNA sequences from bacteria in the last decade. Reports that have shown genetic reference to *Ugba* include the review [16] of relevant *Bacillus* species that can be biotechnologically relevant, the 16S rRNA characterization of lactic acid bacteria [17] and genomic profiling of probiotic *Lactobacilli* [18]. The benefits of increased microbiome profiling of the food condiment have been reiterated [19].

The problem with a few genetic analyses is not only with *Ugba* because several African fermented products are obtained from other oilseeds, but only a few have been investigated for their microbial content holistically [20]. Hence, this investigation aimed to establish the phylogeny and ancestry of 16S RNA fragments found in *Ugba* seeds obtained from ten markets in South-Eastern Nigeria. This may help in the surveillance of deleterious bacteria that occur in the product by regulatory authorities. It will equally be helpful for investigation during a food poisoning outbreak.

## Methods

### Sample and strain collection

Fermented sliced samples of *Ugba* were obtained from 10 different markets. The study did not involve human or animal subjects. Twenty-five grams of the food condiment were dissolved with 225 of maximum recovery diluent (pH=7.01). The pH of the mixture was taken and then incubated for 24 h. A 100 µl of the blended condiment was placed on polymyxin egg yolk mannitol bromothymol blue agar (PEMBA) (PO5048A) medium (Thermo Scientific^™^ Oxoid^™^) and spread out before being incubated at 37° for 24 h using sterile distilled water as a control. Colonies (up to five loopfuls) that emerged were scraped off into Eppendorf tubes and suspended using 1 ml of DNA/RNA Shield (Zymo Research, USA) and then stored at 20 °C for 24 h as per the manufacturer’s instructions.

### DNA Extraction

Forty microlitres of the cell suspension were lysed with 120 µl of TE buffer containing lysozyme (final concentration, 0.1 mg ml^−1^) and RNase A (ITW Reagents, Barcelona, Spain) (final concentration 0.1 mg ml^−1^) and incubated for 25 min at 37 °C. Proteinase K (VWR Chemicals, OH, USA) (final concentration 0.1 mg ml^−1^) and SDS (Sigma-Aldrich, MO, USA) (final concentration 0.5% v/v) were added and then incubated for 5 min at 65 °C, after which the genomic DNA obtained was purified using an equal volume of SPRI beads and resuspended in EB buffer (QIAGEN, Germany). DNA quality (0.2–100 ng) was verified with the Quant-iT dsDNA HS kit (Thermo Fisher Scientific) assay in an Eppendorf AF2200 plate reader (Eppendorf UK Ltd, UK).

### Illumina sequencing

Genomic DNA libraries were prepared using the Nextera XT Library Prep Kit (Illumina, San Diego, USA) following the manufacturer’s protocol. The input DNA was increased twofold, and the PCR elongation time was set to 45 s. DNA quantification and library preparation were carried out on a Hamilton Microlab STAR automated liquid handling system (Hamilton Bonaduz AG, Switzerland). Pooled libraries were quantified using the Kapa Biosystems Library Quantification Kit for Illumina. Libraries were sequenced using Illumina sequencers (HiSeq/NovaSeq) using a 250-bp paired-end protocol. Reads obtained were adapter-trimmed using Trimmomatic 0.30 with a sliding window quality cut-off of Q15 [21]. *De novo* assembly was performed on samples using SPAdes version 3.7 [22], and contigs were annotated using Prokka 1.11 [23]. The 16S rRNA gene sequences detected were used to perform a blast search [24] to identify harbouring strains at the species level. Details of the 35 strains containing these sequences were deposited in GenBank under new accession numbers PQ865836–PQ865870.

### Evolutionary phylogeny and ancestral analysis

This was performed *in silico* with Molecular Evolutionary Genetic Analysis (mega 12) using default settings [25]. Sequences were imported into the mega software and aligned with the multiple sequence alignment (muscle) program [26]. The evolutionary history and the ancestry were inferred using the maximum-likelihood method.

## Results and discussion

### Bacteria harbouring detected 16S rRNA sequences

The pH of samples ranged from 5.57±0.02 to 7.59±0.06, and the sample (sample 3), which had the most fragments of different species, had an acidic pH of 5.71. Overall, 35 16S rRNA fragments found in the *Ugba* samples belonged to 13 species (Table 1). The genus *Proteus* was found in all samples. The predominant species was *Proteus mirabilis* with 19 sequences, followed by *Klebsiella pneumoniae* and *Enterococcus faecalis* with four and two, respectively. Two genera, namely *Proteus* and *Enterococcus,* had sequences from two or more species. The *Proteus* species included *P. mirabilis* and *Proteus columbae*, while that of *Enterococcus included E. faecalis*, *Enterococcus gallinarum* and *Enterococcus casseliflavus* among other species (Table 1). The limitations of the statistics include the lack of quantitative diversity characterization or comparative statistical analysis of the sequences. This initial analysis will form the foundation of future genetic research that will focus on whole-genome sequences.

**Table 1.** Organisms and strains isolated from different samples of *Ugba* in this study

	Strain	Organism	Source and country of closest relative (Source: Genbank)
	***Sample 1***		
1	UGB1NG1	*Proteus mirabilis*	Animal waste; China
2	UGB1NG2	*Proteus mirabilis*	Gastropods under a mangrove tree; India
3	UGB1NG3	*Proteus mirabilis*	Human faeces; UK
4	UGB1NG4	*Klebsiella pneumoniae*	Human blood; Taiwan
	***Sample 2***		
5	UGB2NG1	*Proteus mirabilis*	Human sputum; Czech Republic
6	UGB2NG2	*Proteus mirabilis*	Human sputum; China
7	UGB2NG3	*Alcaligenes nematophilus*	Industrial wastewater; Switzerland
8	UGB2NG4	*Lysinibacillus sphaericus*	16S RNA; India
	***Sample 3***		
9	UGB3NG1	*Klebsiella pneumoniae*	Tracheal aspirate; Russia
10	UGB3NG2	*Klebsiella pneumoniae*	Pig manure; China
11	UGB3NG3	*Klebsiella pneumoniae*	Human gut; Spain
12	UGB3NG4	*Proteus mirabilis*	Duck intestine; China
13	UGB3NG5	*Chryseobacterium bernardetii*	Human DNA; UK
14	UGB3NG6	*Stenotrophomonas geniculata*	Asian citrus psyllid; USA
15	UGB3NG7	*Acinetobacter bereziniae*	Human urine; UK
	***Sample 4***		
16	UGB4NG1	*Proteus mirabilis*	Human sputum; China
	***Sample 5***		
17	UGB5NG1	*Proteus mirabilis*	Human sputum; China
18	UGB5NG2	*Proteus mirabilis*	Human sputum; China
19	UGB5NG3	*Proteus mirabilis*	Human sputum; China
20	UGB5NG4	*Mammaliicoccus sciuri*	Duck viscera; China
	***Sample 6***		
21	UGB6NG1	*Proteus mirabilis*	Clinical; China
22	UGB6NG2	*Proteus mirabilis*	Human sputum; China
23	UGB6NG3	*Proteus mirabilis*	Human sputum; China
24	UGB6NG4	*Enterococcus gallinarum*	Human faeces; China
	***Sample 7***		
25	UGB7NG1	*Proteus mirabilis*	Human DNA; Germany
26	UGB7NG2	*Proteus mirabilis*	Human DNA; Germany
27	UGB7NG3	*Proteus mirabilis*	Human sputum; Czech Republic
28	UGB7NG4	*Enterococcus casseliflavus*	Cloaca from *Gallus gallus*; Germany
	***Sample 8***		
29	UGB8NG1	*Proteus mirabilis*	Human sputum; China
30	UGB8NG2	*Proteus mirabilis*	Human sputum; China
31	UGB8NG3	*Kurthia gibsonii*	Water; China
	***Sample 9***		
32	UGB9NG1	*Proteus mirabilis*	Human sputum; China
	***Sample 10***		
33	UGB10NG1	*Enterococcus faecalis*	Pet food; Switzerland
34	UGB10NG2	*Enterococcus faecalis*	Faecal matter from *Gallus gallus* domesticus; Canada
35	UGB10NG3	*Proteus columbae*	16S RNA; China

The study was initially set up to study *Bacillus cereus* strains that are present in *Ugba*, which is why PEMBA selective medium, which is selective for the bacterium, was used. When other colonies (e.g. swarms of *Proteus*) were observed to grow on it, sequencing was carried out on the mixed colonies that emerged. A common knowledge is that *P. mirabilis* has the propensity to swarm on a wide variety of media and interfere with the identification of other species in a sample. The information provided by the PEMBA media manufacturers states that the limitation of the media is that polymyxin B-resistant organisms will grow on it. *P. mirabilis* is polymyxin B-resistant. The resistance of the organism to polymyxin is widely reported in literature [27,28], and it is believed that one way this resistance is achieved is by mutation of the antibiotic-hydrolysing enzymes, which ensures that antibiotics do not reach the binding site [29]. In the future, a good way to prevent *Proteus* proliferation and competition would be by adding cysteine-lactose-electrolyte-deficient medium to the broth samples because it is known to prevent the swarming phenomenon [30,31].

Information from other Genbank submissions (PQ865836–PQ865870) showed that bacteria harbouring the sequences studied have been isolated from a wide variety of sources in different countries around the world (Table 1). These sources include plants and animals and the main non-living components of ecological systems like water and soil. The dominant *P. mirabilis* has been isolated from clinical, environmental and food samples [32] and isolated from *Ugba* by others. Four out of the 13 species identified in this study have been identified by Okorie *et al*. [33] when they used cultural and molecular techniques to probe enteric pathogens in *Ugba*. The species they identified, among others, include *P. mirabilis*, *E. casseliflavus*, *E. faecalis* and *K. pneumoniae*. The organism, *K. pneumoniae*, is prevalent in foods found in European countries when a multicentre study [34] was carried out. The genus *Acinetobacter* was isolated in our study and theirs. While they isolated *A. baumannii*, our investigation detected *Acinetobacter bereziniae*. Like this study, their molecular techniques did not detect any *Bacillus* species. Although *Bacillus* is believed to be prevalent in *Ugba*, it was possibly outcompeted by *P. mirabilis* and other bacteria in the media used. If a non-selective medium is used, various species of *Bacillus* would be detected.

Other bacteria that have been isolated by others include the species of *Staphylococcus*, *Micrococcus*, *Lactobacillus* and other *Enterobacteriacea*e [35], while another study [36] also isolated *Bacillus* and *Staphylococcus* species. An analysis [37] of *Ugba* fermented in the laboratory yielded *Lysinibacillus xylanilyticus*, whereas this study detected *Lysinibacillus sphaericus*. To the best of the knowledge of the authors, this is the first time that *L. sphaericus*, *A. bereziniae, P. columbae* and *Alcaligenes nematophilus, Chryseobacterium bernardetii*, *Stenotrophomonas geniculata*, *Enterococcus gallinarum*, *Mammaliicoccus sciuri* and *Kurthia gibsonii* have been detected in *Ugba*.

#### Significance of bacteria harbouring detected sequences

Bacteria that occur in foods will not normally cause any health concerns if the numbers are controlled. Some may be part of the natural flora and may cause only spoilage, whereas others get into the food through process contamination and may become pathogenic, especially if the numbers increase. In this study, several strains of *P. mirabilis* were detected. When it is found in food, it suggests that the food was not prepared or stored properly and may be contaminated with faecal residues [38]. The clinical significance of *P. mirabilis* is well known because it has been associated with several infections in humans. These infections include appendicitis, foodborne and spontaneous gastroenteritis [39], urinary tract [40] and surgical site [41] infections. The pathogenicity of the other *Proteus* species (*P. columbae*) detected in another study [42] is yet to be fully determined.

*K. pneumoniae* is prevalent in meat and raw food [42,44]. The bacteria are now a global concern because they can cause septicemia, urinary tract infections, pneumonia, meningitis, wound burn, surgical site infections and liver abscesses in both the elderly and young people [45]. Multidrug-resistant strains have been found to overlap among isolates from food and humans [46]. Another species of clinical importance detected in this study is the genus *Enterococcus*. The species *E. faecalis* may occur in ready-to-eat food [47] and occurs in the gastrointestinal tract of animals. The bacterium is known for its antibiotic resistance and can be used as an indicator of faecal contamination. Several infections like peritonitis, endocarditis and bacteremia have been associated with the bacteria.

The properties of the other *Enterococcus* species, namely *E. gallinarum* and *E*. *casseliflavus*, have been reviewed. In the review [48], it was highlighted that both are vancomycin-resistant. Also, *E. gallinarum* may be found in fish, meat and cheese, and even though it occasionally occurs in the gastrointestinal tract, it is not considered an indicator of faecal contamination. Another highlight was that *E. casseliflavus* has been observed in forage plants and implicated with *E. gallinarum* in causing health infections in the urinary tract, bloodstream and surgical wounds.

The other species detected in the study do not cause infections on the scale of the aforementioned species. *A. nematophilus* is an environmental contaminant and can cause gastroenteritis with the help of antibiotic resistance genes it harbours [49]. Also, *Acinetobacter bereziniae* is regarded as an opportunistic pathogen that possesses multidrug resistance genes, and it has been reported to cause infections in hospitals. The bacteria have been isolated from human milk [50]. Furthermore, *Chryseobacterium* species can cause nosocomial infections [51] while *Mammaliicoccus sciuri*, previously classified as an occasional pathogen, possesses resistance and virulence transfer genes [52]. The bacteria *K. gibsonii* may be found in milk and meat products and can produce antimicrobial peptides [53], but information on its genomes is limited [54]. *L. sphaericus* is found in the environment and has been used to control malaria and other tropical diseases [55], while *S. geniculata* is an endophyte that can live in plant tissues and can be used to control postharvest fungi [56]. It is possible that the two bacteria mentioned found their way into *Ugba* from the field.

While there are pathogenic concerns about *Ugba*, fermented foods from African regions contain non-pathogenic micro-organisms like *Lactobacillus* species, which can utilize substrates to produce useful metabolites [57]. There are also indications that diverse non-pathogenic strains from fermented foods can resolve gut problems, improve colon health and exert a probiotic effect [58]. This suggests that fermented *Ugba* may indeed possess the beneficial health and probiotic effects [5] earlier reported. However, there is a concern that well-established fermentation knowledge applicable to cheese and wine has yet to be applied to new matrices for innovation and new product development [59]. Hence, there is an opportunity for further research to unravel the metabolic pathways of pathogenic and non-pathogenic micro-organisms of the food product.

### Evolutionary phylogeny of sequences

**Fig. 1.**
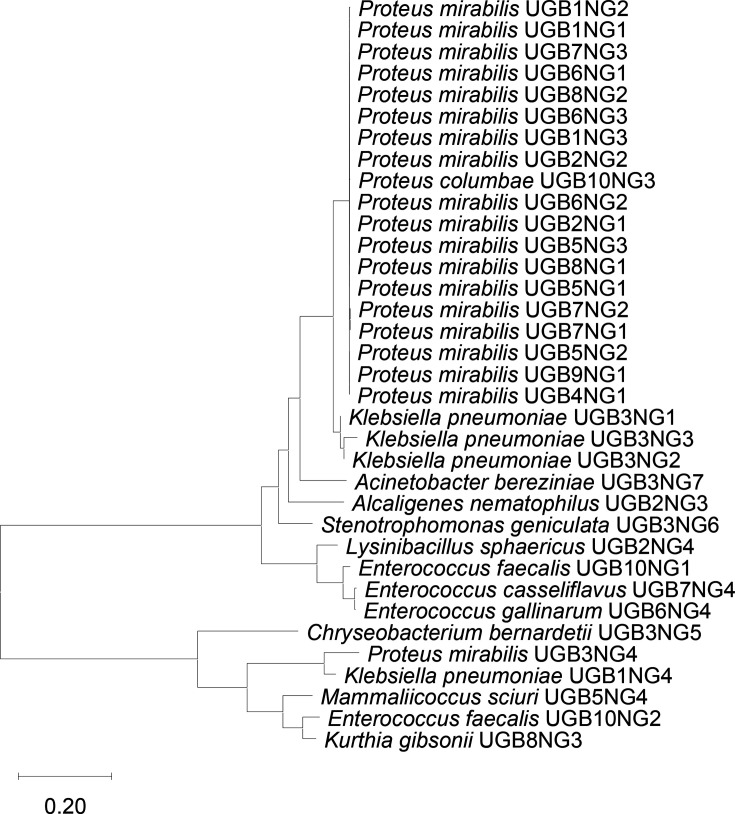
Evolutionary analysis by the maximum-likelihood method: The phylogeny was inferred using the maximum-likelihood method model of nucleotide substitutions and the tree with the highest log-likelihood. The analytical procedure encompassed 35 taxons with 1,857 positions in the final dataset. Evolutionary analysis was conducted in mega12 [21].

The only *P. columbae* detected and all the *P. mirabilis* strains arose from one node, apart from strain UGB3NG4, which was found in a different clade (Fig. 1). This indicates that most of the *P. mirabilis* detected may be the same clones. All the *K. pneumoniae* strains were in the same monophyletic group apart from strain UGB1NG4, while the two *E. faecalis* strains did not originate from the same node (Fig. 1). The strain UGB10NG1 formed a clade with other *Enterococcus* genera, while UGB10NG2 fell under a monophyletic group with other bacteria. The pattern observed in *P. mirabilis, K. pneumoniae* and *E. faecalis* suggests at least two distinct lineages or species divergence. The species *C. bernardetii* did not share any relationship with any other bacteria. When the overall phylogeny is considered, it appears that most of *P. mirabilis* got into the samples much later, which suggests processing contamination.

The evolutionary phylogeny for different *Ugba* bacteria is yet to be reported. The phylogeny of the main *Enterobacteriaceae* bacteria detected in this study from other sources is well studied. A report [60] pointed out that *P. mirabilis* can be divided into three subspecies and that subspecies 1 represents 97% of all genomes and consists of related clonal groups. Another comprehensive analysis [61] of over 3,700 genomes from 58 countries ascertained that there were 17 distinct clusters. Furthermore, a proposal [62] to use core genome multilocus sequence typing as a method to identify clonal groups (CGs) in *P. mirabilis* found 205, which were influenced by regional differences. If whole-genome sequences of the strains are obtained in the future, it will help establish what their subspecies, clusters and CGs are.

For *E. faecalis*, it has been pointed out [63] that there are two distinct lineages based on whole-genome sequencing studies. These include lineage (clade A), which is hospital-associated, and lineage (clade B), which is community-associated. Considering that the two strains detected in this study resolved into different clades, we posit that one may be lineage clade A and the other lineage clade B. Further work is required to establish the true lineages. The genetic diversity of other species in this study (*K. pneumoniae*), which showed multiple clones, has also been studied by others. A study [64] clustered over 16,000 genomes obtained from around the world and found that up to 60 clusters were established. The data helped to trace the global route of the evolution of *K. pneumoniae*. It was worrying that 52% of the strains traced harboured genes associated with carbapenem resistance. It would be interesting to investigate and establish which cluster the four strains detected in this study belong to and ascertain if they are carbapenem-resistant.

### Ancestry of sequences

**Fig. 2.**
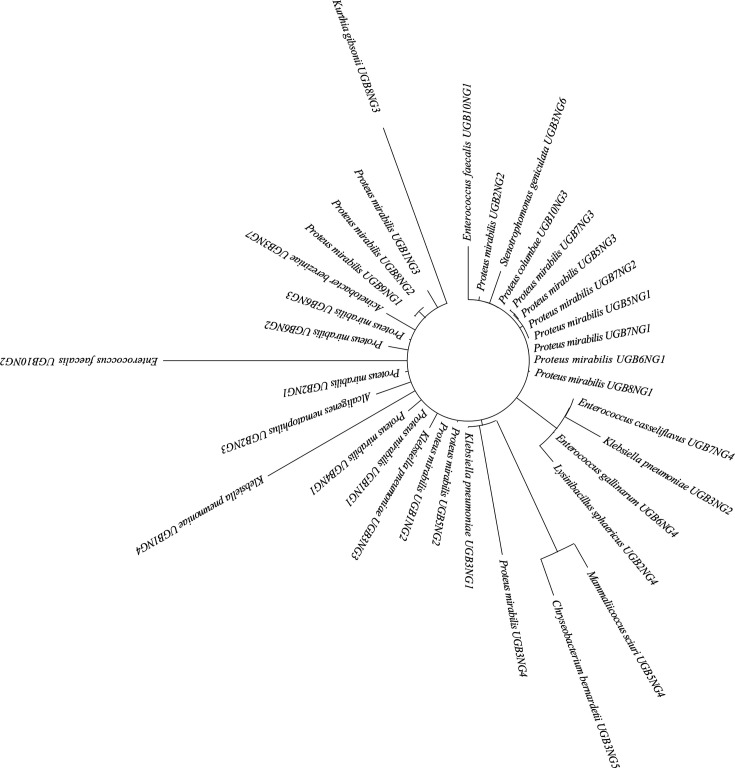
Inferred ancestral sequences: ancestral states were inferred using the maximum-likelihood method. The tree shows a set of possible nucleotides (states) at each ancestral node based on their inferred likelihood. The analytical procedure encompassed 35 nucleotide sequences with 1,857 positions in the final dataset.

The species *K. gibsonii* was found to be the earliest ancestor following ancestral sequence analysis and was followed by a *P. mirabilis* strain UGB7NG3 (Fig. 2). This suggests that *P. mirabilis* may occur at various points in the processing of the product. *K. gibsonii* occurs in the environment, and it got into the sample during harvest from the field, as pointed out earlier. The *K. pneumoniae* strain UGB1NG4 had a longer branch length than other *K. pneumoniae* strains, and this could mean that it occurred in *Ugba* samples first.

The point at which different bacteria got into the seeds is unknown; hence, it may be beneficial to conduct a farm-to-fork study to determine species present when the seeds were in the field, at harvest, during processing and storage until it is finally packaged for sale. The ancestry and succession of bacterial species and the mechanisms that drive microbial succession in *Ugba* are yet to be well studied. A study [65] on the succession of bacteria during the fermentation of *Ugba* for 3 days found that up to 10 bacterial species were present at the beginning, but only *Bacillus* and *Lactobacillus* species were present after 72 h. The bacteria that were present at the beginning included *Staphylococcus aureus*, *Micrococcus*, *Escherichia coli* and *Klebsiella* species. Other species were *Streptococcus*, *Pseudomonas*, *Proteus* and *Salmonella*.

Food that is fermented is now described as ‘made through desired microbial growth and enzymatic conversions of food components’ [66] and a lot of factors have been found to influence succession. A report highlighted [67] that differences in the assembly of microbiota may be influenced by climate conditions, geographical location, agricultural practices and processing after harvest. Despite these differences, the microbial content and their primary metabolic functions in natural fermentations are consistent. Hence, it can be concluded that the ancestral microbiome of *Ugba* in the last century has not changed much since the same flavour and texture after fermentation by microbes has remained unchanged for decades.

## Conclusions

Bacteria of food safety concern were found to be present in fermented *Ugba* seeds, and there may be distinct evolutionary lineages present in samples from different environments. Some of the species detected have been clinically proven to be pathogens; hence, a pretreatment of the product to eliminate pathogenic bacteria before sale may help prevent food poisoning outbreaks. The ancestry of the sequences in the products studied showed that the species *K. gibsonii* was the earliest ancestor. Considering that species that have not been previously associated with the product were detected, more work is required to ascertain the microbial dynamics and their metabolic products in different environments. This will help to establish the *Ugba* fermentation aspects that are relevant to pathogenic and non-pathogenic food safety concerns.

## Abbreviations

CGs: clonal groups
mega: Molecular Evolutionary Genetic Analysis
PEMBA: polymyxin egg yolk mannitol bromothymol blue agar

## References

[1] Okafor GI, Okpala LC, Uchegbu NN, Anchang MM, Aworh OC, Owusu-Darko PG, Lelieveld H, Andersen V, Prakash V (2024). Nutritional and Health Aspects of Food in Western Africa [Internet].

[2] Ogueke CC, Nwosu JN, Owuamanam CI, Iwouno JN (2010). *Ugba*, the fermented african oilbean seeds; its production, chemical composition, preservation, safety and health benefits. Pak J Biol Sci.

[3] Ogueke CC, Anosike F, Owuamanam CI (2015). Prediction of amino nitrogen during *Ugba* (*Pentaclethra macrophylla*) production under different fermentation variables: a response surface approach. Niger Food J.

[4] Omonhinmin CA, Alonge KO (2024). Intra-specific genetic diversity, phylogenetic analysis and ecological preferences of *Pentaclethra macrophylla* Benth., across Nigeria based on rbcL dataset. Data Brief.

[5] Anosike FC, Onyemah KO, Ossai CU, G Ofoegbu JN, Okpaga FO (2022). Probiotic potential and viability of bacteria in fermented African oil bean seed (*Pentaclethra macropyhlla*): a mini review. Appl Food Res.

[6] Oranusi S, Braide W, Chinakwe E, Department of Microbiology, Federal University of Technology, Owerri, Imo State, Nigeria (2013). Probiotic carrier potential, sensory properties and microbial quality of *Ugba* (*Pentaclethra macrophylla*) and ogiri (*Ricinus communis*). Int J of Micr Res.

[7] Alo M, Ugah U, Ndie E, Ukwa B, Ilang D (2014). Probiotic potential of nigerian fermented food *Pentaclethra macrophylla* (*Ugba*). World J Public Health Sci.

[8] Ilango S, Antony U (2021). Probiotic microorganisms from non-dairy traditional fermented foods. Trends Food Sci Technol.

[9] Ugbogu EA, Nwoku CD, Ude VC, Emmanuel O (2020). Evaluating bioactive constituents and toxicological effects of aqueous extract of fermented *Pentaclethra macrophylla* seeds in rats. Avicenna J Phytomed.

[10] Anowu JN, Aliyu AB, Ibrahim H, Oyewale AO (2021). Isolation of bioactive compounds from the fermented seeds of *Pentaclethra macrophylla*. Chem Nat Compd.

[11] Afia K (2020). A review of *Pentaclethra macrophylla* (African oil bean) seed. IDOSR J Exp Sci.

[12] Okiemute Rosa JA, Jesam U (2024). Evaluation of the healing properties of pentaclethra macrophylla seed pod on diabetic wounds. Nig J Pharm Appl Sci Res.

[13] Nwahiri JD, Tamuno-Emine DG, Nwachuku EO, Bartimaeus ES (2021). Antioxidant potentials of *Pentaclethra macrophylla*
seed (*Ugba*) on mercury toxicity induced hepatic, renal and testicular oxidative stress in male albino rats. J Complement Altern Med Res.

[14] Agunloye OM, Oboh G (2023). Fermented seeds of *Pentaclethra macrophylla* mitigate against memory deficit and restored altered enzymatic activity in the brain of streptozotocin-diabetic rats. Metab Brain Dis.

[15] Ogueri N The good and bad of the African oil bean (*Pentaclethra macrophylla*) seed, *Ugba*: a perspective on its property as a delicious delicacy or a death agent. Scholars virtual meeting, January 2025. p. 1–4. https://www.researchgate.net/publication/388173384.

[16] Owusu-Kwarteng J, Parkouda C, Adewumi GA, Ouoba LII, Jespersen L (2022). Technologically relevant *Bacillus* species and microbial safety of West African traditional alkaline fermented seed condiments. Crit Rev Food Sci Nutr.

[17] Ogunleke OB, Oladipo IC (2025). Isolation and molecular characterization of lactic acid bacteria isolated from selected fermented food condiments. World J Bio Pharm Health Sci.

[18] Obafemi Y, Oranusi S, Oluseyi AK, Akinduti P (2022). Genotyping of probiotic *Lactobacilli* in Nigerian fermented condiments for improved food safety. Open Access Maced J Med Sci.

[19] Obafemi YD, Oranusi SU, Ajanaku KO, Akinduti PA, Leech J (2022). African fermented foods: overview, emerging benefits, and novel approaches to microbiome profiling. NPJ Sci Food.

[20] Chinma CE, Ezeocha VC, Adedeji OE, Inyang CU, Enujiugha VN, Adebo OA, Chinma CE, Obadina AO, Soares AG, Panda SK (2023). Indigenous Fermented Foods for the Tropics.

[21] Bolger AM, Lohse M, Usadel B (2014). Trimmomatic: a flexible trimmer for illumina sequence data. Bioinformatics.

[22] Bankevich A, Nurk S, Antipov D, Gurevich AA, Dvorkin M (2012). SPAdes: a new genome assembly algorithm and its applications to single-cell sequencing. J Comput Biol.

[23] Seemann T (2014). Prokka: rapid prokaryotic genome annotation. Bioinformatics.

[24] Zhang Z, Schwartz S, Wagner L, Miller W (2000). A greedy algorithm for aligning DNA sequences. J Comput Biol.

[25] Kumar S, Stecher G, Suleski M, Sanderford M, Sharma S (2024). MEGA12: molecular evolutionary genetic analysis version 12 for adaptive and green computing. Mol Biol Evol.

[26] Edgar RC (2004). MUSCLE: multiple sequence alignment with high accuracy and high throughput. Nucleic Acids Res.

[27] Sud IJ, Feingold DS (1970). Mechanism of polymyxin B resistance in *Proteus mirabilis*. J Bacteriol.

[28] Olaitan AO, Morand S, Rolain J-M (2014). Mechanisms of polymyxin resistance: acquired and intrinsic resistance in bacteria. Front Microbiol.

[29] Alqurashi E, Elbanna K, Ahmad I, Abulreesh HH (2022). Antibiotic resistance in *Proteus mirabilis*: mechanism, status, and public health significance. J Pure Appl Microbiol.

[30] Maldonado-Barrueco A, Grandioso-Vas D, Rico-Nieto A, García-Rodríguez J, García-Bujalance S (2022). Closing brief case: *Proteus mirabilis* causing coraliform lithiasis and bacteremia in an elderly catheterized patient. J Clin Microbiol.

[31] Pearson MM, Pearson MM (2019). Proteus Mirabilis: Methods and Protocols.

[32] Chowdhury G, Majumdar T, Modi D, Dolma KG, Chaliha Hazarika S (2025). Description on the prevalence of *Proteus mirabilis* through an integrated sampling framework for health, food, and environment in Northeast India and an integrative review with reference to one health context. Front Microbiol.

[33] Okorie PC, Anike FN, Elemo GN, Isikhuemhen OS (2017). Incidence of enteric pathogens in *Ugba*, a traditional fermented food from African oil bean seeds (*Pentaclethra macrophylla*). Int J Food Contam.

[34] Rodrigues C, Hauser K, Cahill N, Ligowska-Marzęta M, Centorotola G (2022). High prevalence of *Klebsiella pneumoniae* in European food products: a multicentric study comparing culture and molecular detection methods. Microbiol Spectr.

[35] Isu NR, Njoku HO (1997). An evaluation of the microflora associated with fermented African oil bean (*Pentaclethra macrophylla* bentham) seeds during *Ugba* production. *Plant Foods Hum Nutr*.

[36] Ogbulie TE, Nsofor CA, Nze FC (2014). Bacteria species associated with *Ugba* (*Pentaclethra macrophylla*) produced traditionally and in the laboratory and the effect of fermentation on product of oligosaccharide hydrolysis. Niger Food J.

[37] Ahaotu I, Anyogu A, Njoku OH, Odu NN, Sutherland JP (2013). Molecular identification and safety of *Bacillus* species involved in the fermentation of African oil beans (*Pentaclethra macrophylla* benth) for production of *Ugba*. *Int J Food Microbiol*.

[38] Kushwaha K, Babu D, Juneja VK, Batt CA, lou TM (2014). Encyclopedia of Food Microbiology.

[39] Hamilton AL, Kamm MA, Ng SC, Morrison M (2018). *Proteus* spp. as putative gastrointestinal pathogens. Clin Microbiol Rev.

[40] Li H, Xue X, Meng G, He C, Tong L (2025). The roles of bacteria on urolithiasis progression and associated compounds. Biochem Pharmacol.

[41] Ekanem E, Ngene NC, Moodley J, Konje J (2023). Prevention of surgical site infection and sepsis in pregnant obese women. Best Pract Res Clin Obstet Gynaecol.

[42] Dai H, Wang Y, Fang Y, Xiao T, Huang Z (2018). *Proteus columbae* sp. nov., isolated from a pigeon in Ma’anshan, China. Int J Syst Evol Microbiol.

[43] Junaid K, Ejaz H, Younas S, Alanazi A, Yasmeen H (2022). Detection of *Klebsiella pneumoniae* antibiotic-resistant genes: an impending source of multidrug resistance dissemination through raw food. Saudi J Biol Sci.

[44] Tang F, Chen Z, Zhu H, Xi L, Li C (2024). Genetic relatedness, antibiotic resistance, and virulence of *Klebsiella pneumoniae* isolated from health care and food sources in Wuhan, China. Am J Infect Control.

[45] Singh AN, Singh A, Singh SK, Nath G (2024). *Klebsiella pneumoniae* infections and phage therapy. Indian J Med Microbiol.

[46] Silva-Bea S, Romero M, Parga A, Fernández J, Mora A (2024). Comparative analysis of multidrug-resistant *Klebsiella pneumoniae* strains of food and human origin reveals overlapping populations. *Int J Food Microbiol*.

[47] Chajęcka-Wierzchowska W, Zadernowska A, García-Solache M (2020). Ready-to-eat dairy products as a source of multidrug-resistant Enterococcus strains: phenotypic and genotypic characteristics. *J Dairy Sci*.

[48] Monticelli J, Knezevich A, Luzzati R, Di Bella S (2018). Clinical management of non-faecium non-faecalis vancomycin-resistant enterococci infection. Focus on *Enterococcus gallinarum* and *Enterococcus casseliflavus*/*flavescens*. *J Infect Chemother*.

[49] David EE, Igwenyi IO, Iroha IR, Martins LF, Uceda-Campos G (2024). First-genome sequence data of an *Alcaligenes nematophilus* strain EBU-23 encoding bla gene implicated in acute childhood gastroenteritis. *Curr Microbiol*.

[50] Reyes SM, Bolettieri E, Allen D, Hay AG (2020). Genome sequences of four strains of *Acinetobacter bereziniae* isolated from human milk pumped with a personal breast pump and hand-washed milk collection supplies. Microbiol Resour Announc.

[51] Chaudhary R, Kar M, Jamwal A, Dubey A, Singh R (2023). Characteristics of *Chryseobacterium bacteremia*, associated risk factors and their antibiotic susceptibility pattern at a university hospital: a descriptive, retrospective study. Access Microbiol.

[52] de Carvalho A, Giambiagi-deMarval M, Rossi CC (2024). *Mammaliicoccus sciuri’s* pan-immune system and the dynamics of horizontal gene transfer among *Staphylococcaceae*: a one-health CRISPR tale. *J Microbiol*.

[53] Chauhan A, Samant SA (2022). Isolation and identification of *Kurthia gibsonii* from paneer and study its antibacterial activity against intestinal pathogens. Int J Health Sci.

[54] Mukhopadhyay BC, Mitra S, Kazi TA, Mandal S, Biswas SR (2019). Draft genome sequence of cold-tolerant *Kurthia gibsonii* B83, isolated from spinach leaf. *Microbiol Resour Announc*.

[55] Hernández-Santana A, Gómez-Garzón C, Dussán J (2022). Lysinibacillus sphaericus. *Trends Microbiol*.

[56] Ling L, Wang Y, Li J, Cheng W, Yue R (2024). Endophytic *Stenotrophomonas geniculata* KJ-6 via producing antifungal volatile organic compounds effectively control *Lanzhou lily* postharvest diseases. Food Biosci.

[57] Chen S, Zhang C, Yin P, Chen R, Yu L (2026). Exploring microbial diversity and quality in fermented foods from tropical and non-tropical regions. Food Nutrition.

[58] Malongane F, Berejena T (2024). Exploring the microbiome present in fermented indigenous African foods and their potential impact on human health. J Agric Food Res.

[59] Roland N, Spatz M, Le-Loir Y, Steyer J-P, Schbath S (2026). Benefits of fermented foods, from empiricism to scientific evidence: the contribution of Ferments du Futur. Cahiers de Nutrition et de Diététique.

[60] Potter RF, Zhang K, Reimler B, Marino J, Muenks CE (2023). Uncharacterized and lineage-specific accessory genes within the *Proteus mirabilis* pan-genome landscape. mSystems.

[61] Zhang S, Li Q, Wang M, Jia R, Chen S (2025). Genomic analysis of *Proteus mirabilis*: unraveling global epidemiology and antimicrobial resistance dissemination - emerging challenges for public health and biosecurity. Environ Int.

[62] Chen SL, Kang YT, Liang YH, Qiu XT, Li ZJ (2023). A core genome multilocus sequence typing scheme for *Proteus mirabilis*. Biomed Environ Sci.

[63] Cattoir V (2022). The multifaceted lifestyle of enterococci: genetic diversity, ecology and risks for public health. Curr Opin Microbiol.

[64] Heng H, Yang X, Ye L, Tang Y, Guo Z (2024). Global genomic profiling of *Klebsiella pneumoniae*: a spatio-temporal population structure analysis. Int J Antimicrob Agents.

[65] Obi CN, Nkemnaso C (2018). Effects of thermal processing and fermentation on African oilbean (*Pentaclethra Macrophylla*) seeds during *Ugba* production. Biosci Bioeng.

[66] Marco ML, Sanders ME, Gänzle M, Arrieta MC, Cotter PD (2021). The International Scientific Association for Probiotics and Prebiotics (ISAPP) consensus statement on fermented foods. Nat Rev Gastroenterol Hepatol.

[67] Auchtung JM, Hallen-Adams HE, Hutkins R (2025). Microbial interactions and ecology in fermented food ecosystems. *Nat Rev Microbiol*.

